# Risk Factors and Predictors of Mortality in Critically ill Children with Extensively-Drug Resistant *Acinetobacter baumannii* Infection in a Pediatric Intensive Care Unit

**Published:** 2014-09-11

**Authors:** Kapil Kapoor, Sumidha Jain, Mamta Jajoo, Swati Dublish, Vikas Dabas, Vikas Manchanda

**Affiliations:** Maulana Azad Medical College and Associated Chacha Nehru Bal Chikitsalaya, Delhi, India

**Keywords:** Acinetobacter Baumannii, Extensively-Drug Resistant, Hospital Associated Infection, Children

## Abstract

***Objective:*** *Acinetobacter baumannii* is an important emerging cause for extensively-drug resistant (XDR) hospital associated infections (HAIs) in pediatric intensive care units (PICU). The study was done to evaluate the risk factors, outcome, antibiotic sensitivity pattern, and predictors of mortality in critically ill children with XDR *A. baumannii* infection.

***Methods:*** Retrospective case control study, done in the PICU of a tertiary care pediatric hospital of India from April 2010 to March 2012.

***Findings:*** Eighty-five children who developed XDR *A. baumannii* infection matched to 170 controls. Majority (76%) of the organisms were isolated from endotracheal lavage. The mortality rate was 28.2% (24/85). The factors found to be significantly associated with *A. baumannii* infection were prior use of broad-spectrum antibiotics, major surgeries done, prolonged PICU stay, use of central venous catheters, and mechanical ventilation. The predictors of mortality associated with *A. baumannii* infection were acute kidney injury, presence of septic shock, and disseminated intravascular coagulopathy. Colistin found to be the single most effective drug against XDR *A. baumannii* infection.

***Conclusion:*** XDR *A. baumannii* infections are associated with high morbidity and mortality in critically ill children. Early diagnosis and treatment are crucial. Implementation of infection control practices and rational use of antibiotics are required to control such infections.

## Introduction

*Acinetobacter baumannii* (*A. baumannii)* is a gram-negative coccobacillus and ubiquitous microorganism that has become an important pathogen for hospital associated infections (HAIs), particularly in intensive care units (ICUs)^[^^1^^,^^2^^]^. Although classically described as a HAIs pathogen in adults, *A. baumannii* is emerging as an important pathogen in children. Its rapid acquisition of a wide variety of antibiotic resistant genes has caused serious therapeutic problems worldwide^[^^3^^]^.

 Nosocomial infection with *A. baumannii* results in pneumonia, bacteraemia, meningitis, and urinary tract infection^[^^4^^,^^5^^]^. Several factors have been associated with *A. baumannii* infection including ICU admission, mechanical ventilation, invasive procedures, and the use of broad-spectrum antimicrobials^[^^6^^]^. The emergence of extensively drug resistant strains poses a great health hazard. 

 The aim of this study was to evaluate the risk factors, outcome, antibiotic sensitivity pattern, and predictors of mortality in critically ill children with *A. baumannii* infection in the Pediatric Intensive Care Unit (PICU) of a tertiary care pediatric hospital.

## Subjects and Methods

This retrospective case control study was conducted in the 12 bedded PICU of Maulana Azad Medical College and associated Chacha Nehru Bal Chikitsalaya, Delhi (India) from April 2010 to March 2012 (two years).

*Inclusion criteria:* A case was defined as any child hospitalized in the PICU during the study period that developed *A. baumannii* infection confirmed by laboratory examination. Other children hospitalized during the same period in the PICU with no diagnosis of infectious syndrome form the controls. The controls were selected at random. 

*Exclusion criteria:* (1) Patients with poly-microbial infection in addition to *A. baumannii* infection; and (2) Patients who left against medical advice (LAMA).

*Data extraction: *An individual record was obtained from the medical health records, which included possible predisposing factors such as age, gender, duration of PICU stay, use of broad-spectrum antibiotics, presence of septic shock and other co-morbid conditions, duration of mechanical ventilation, use of a central or peripheral venous catheter, urinary catheters, and major surgical procedures if any. 

*A. baumannii* infection was diagnosed in patients with clinical evidence of infection with one or more isolates of *A. baumannii* from clinical sample.

 Standard definitions for HAIs used according to the Centre for Disease Control and Prevention^[^^7^^]^.

 Pneumonia defined if there was isolation of *A. baumannii* from pulmonary secretions with concurrent infiltrates on chest radiography and clinical signs and symptoms of infection.

 Ventilator-associated pneumonia (VAP) diagnosed based on Centre for Disease Control and Prevention clinical, radiographic, and microbiologic criteria^[^^7^^]^.

 All children were diagnosed with AKI based on Acute Kidney Injury Network (AKIN) criteria^[^^8^^]^.

 Septic shock defined as the presence of sepsis and cardiovascular organ dysfunction^[^^9^^]^.

 The *A. baumannii* isolates divided into the following drug resistance categories^[^^10^^]^:

*Multi-drug resistant (MDR)* defined as acquired non-susceptibility to at least one agent in three or more antimicrobial categories (namely, aminoglycosides, antipseudomonal carbapenems, antipseudomonal fluoroquinolones, antipseudomonal penicillins+β-lactamase inhibitors, extended-spectrum cephalosporins, folate pathway inhibitors, penicillins+β-lactamase inhibitors, polymyxins, tetracyclines)

*Extensively drug resistant (XDR) *defined as non-susceptibility to at least one agent in all but two or fewer antimicrobial categories (i.e. bacterial isolates remain susceptible to only one or two categories).

*Pan-drug resistant (PDR) *defined as non-susceptibility to all agents in all antimicrobial categories.

 Blood cultures obtained in Bactec culture bottles, other sterile body fluids obtained in sterile containers before starting antibiotics in symptomatic cases, and subcultures were done on MacConkey's medium for further colony growth. The organism was identified by the Vitek-2 compact system. Antimicrobial susceptibility to colistin tested with the disk diffusion method following Clinical Laboratory Standards Institute guidelines^[^^11^^]^. Repeat cultures were taken when antibiotics changed due to poor response. In vitro sensitivities were carried out using disc method for the following antibiotics: Imipenem, meropenem, piperacillin-sulbactam, colistin, trimethoprim-sulphamethoxazole, chloramphenicol, amikacin, gentamicin, netilmicin, ceftriaxone, cefotaxime, cefepime, ceftazidime, ciprofloxacin, levofloxacin, and ampicillin-sulbactum.

 The primary outcome was all cause mortality (death at any time during the PICU admission) and secondary outcomes were risk factors for Acinetobacter infection, antimicrobial resistance and their sensitivity pattern and the predictors of mortality. 

**Table 1:** Characteristics and risk factors of the study group

**Parameter**	**Cases** **(n=85)**	**Controls** **(n=170)**	***P*** **-value** ^*^
**Age (months) (mean±standard deviation)**	24.6 (36.4)	28.2 (34.7)	0.6
**Male : Female**	56:29	92:78	0.07
**Previous Hospitalization**	32	43	0.06
**Major Surgeries**	06	01	0.006
**Prior use of Broad Spectrum Antibiotics**	84	152	0.003
**PICU stay (days) (mean ± standard deviation)**	25.3 (13.7)	13.4 (9.7)	0.0001
**Use of Central Venous Catheters**	79	127	0.0002
**Mechanical Ventilation required**	78	119	0.00004
**Urinary Catheters**	84	161	0.1
**Intercostal Drainage tubes**	6	17	0.5

 Categorical variables were compared by using the likelihood ratio test or, when appropriate, Fisher's exact test. The Student’s t-test or the Wilcoxon Rank-Sum Test for non-parametric distributions analyzed continuous variables. All tests were two-tailed with *P*<0.05 considered significant.

## Findings

During the study period, there were 1022 admissions in the PICU. Out of these, 90 children had symptomatic *A. baumannii* infection. Of these (90), three patients went LAMA and two grew other bacteria and hence excluded from the study. Finally, 85 (8.3%) infected children formed the case group and compared with 170 matched controls. The study patients' characteristics and risk factors are shown in Table 1. 

 The factors found to be significantly associated with *A. baumannii* infection were prior use of broad-spectrum antibiotics, major surgeries done, prolonged PICU stay, use of central venous catheters and mechanical ventilation, when compared to the control group (*P*<0.05). 

 In the case group, primary diagnosis at admission was pneumonia (72.9%), bloodstream infection (21.1%), meningitis (3.5%), and empyema thoracis (2.3%). Six children had undergone major surgeries for posterior mediastinal teratoma, esophageal stricture, left lobectomy with congenital cystic adenoid malformation, ileal perforation, intestinal perforation, and jejunal atresia.

*A. baumannii* was isolated from 92 cultures in 85 patients. Majority (76%) of the organisms isolated from endotracheal lavage (Table 2). In our study majority (79/85) of *A. baumannii* isolates were XDR, and six were MDR. Empirical antibiotic therapy given to all according to hospital antibiotic policy, later antibiotic was changed according to culture sensitivity. The mean duration of antibiotics before Acinetobacter culture positivity was 17.4±9.3 days. 

 The strains of *A. baumannii* found to be highly resistant to several of the antimicrobial drugs examined (Table 3). In vitro, antimicrobial susceptibility showed 95.6% (88/92) sensitivity to colistin. Only 5.5% were sensitive to carbepenems. Piperacillin-sulbactam, ciprofloxacin and gentamicin showed 3.2% susceptibility each. Third and fourth generation cephalosporins showed 100% resistance. The mortality rate in surgical patients was 50% (3/6). The overall mortality was 28.2 % (24/85).

**Table 2:** *A. baumannii* isolation sites

**Site**	**Number (n=92)**	**%**
**Endotracheal lavage**	70	76
**Blood**	15	16.4
**Urine**	5	5.5
**Pleural fluid**	2	2.1

**Table 3:** Antimicrobial susceptibility of *A. baumannii* isolates

**Drugs**	**Sensitive**	**Intermediate ** **sensitive**	**Resistant** **(%)**
**Ampicillin-sulbactum **	0	0	92 (100%)
**Ceftriaxone **	0	0	92 (100%)
**Cefotaxime **	0	0	92 (100%)
**Cefipime **	0	0	92 (100%)
**Ceftazidime **	0	0	92 (100%)
**Netilmicin**	0	0	92 (100%)
**Levofloxacin **	0	0	92 (100%)
**Trimethoprim-sulphamethoxazole**	1	0	91 (98.9%)
**Chloramphenicol **	1	0	91 (98.9%)
**Ciprofloxacin **	2	1	89 (96.7%)
**Gentamicin **	2	1	89 (96.7%)
**Piperacillin-sulbactum **	2	1	89 (96.7%)
**Imipenem **	2	3	87 (94.5%)
**Meropenem **	3	2	87 (94.5%)
**Colistin **	92	0	0

The predictors of mortality associated with *A. baumannii* infection were acute kidney injury, presence of septic shock, and disseminated intravascular coagulopathy (DIC) (Table 4).

## Discussion

Acinetobacter species have become an important culprit in HAIs and in recent years, have displayed increasing resistance to a broad range of antimicrobials^[^^12^^]^. Even though outbreaks caused by *A. baumannii* described in medical and surgical wards, ICUs are the most frequently affected areas as patients admitted in ICUs usually need more invasive procedures for longer periods, and frequently receive treatment with antimicrobials^[^^13^^]^.

 We reported a mortality rate of 28.2% ( 24/85). Previous studies have reported mortality ranging from 17% to 63%^[^^14^^-^^17^^]^. Mortality rates were higher (50%) in surgical patients, which may be due to the prolonged ventilator support required by these patients and the post-operative broad-spectrum use of antibiotics. Pneumonia was the most common clinical presentation in our study, similar to other reports^[^^15^^,^^18^^]^.

**Table 4:** Characteristics of patients with *A. baumannii* infection

**Parameter**	**Survived** **(n=61)**	**Expired** **(n=24)**	***P*** **-value**
**Age(months) (mean±standard deviation)**	25.3 (34.1)	23.2 (35.4)	0.9
**Age ≤1 year**	18	31	0.05
**Male:Female**	14:10	42:19	0.4
**Previous hospitalization (n=32)**	11	21	0.3
**Major surgeries (n=6)**	03	03	0.3
**Prior use of broad spectrum antibiotics (n=84)**	24	60	0.7
**PICU stay(days) (mean±standard deviation)**	28.3 (14.5)	20.1 (10.4.)	0.4
**Use of central venous catheters (n=79)**	23	56	0.6
**Mechanical ventilation required (n=78)**	24	54	0.2
**Shock (n=58)**	23	35	0.0002
**Disseminated intravascular coagulopathy (DIC)**	13	11	0.001
**Acute kidney Injury**	7	4	0.01

 The known risk factors for *A. baumannii *infection are invasive procedures and the use of broad-spectrum antimicrobials^[^^13^^]^. In consistence to these, we found major surgeries done, use of central venous catheters, mechanical ventilation, and use of broad-spectrum antibiotics to be statistically significant risk factors. However, use of urinary catheters and intercostal drainage tubes were not statistically associated with Acinetobacter infection as shown by others^[^^18^^]^. Acinetobacter has the ability to survive for long

periods on inanimate surfaces in the patient’s vicinity thereby providing a constant source of infection ^[^^19^^]^. Longer the PICU stay, the more the exposure and hence this has been reported as a risk factor as in other studies^[^^20^^,^^21^^]^. 

 Previous studies have shown risk factors associated independently with poor prognosis being severity of the underlying disease, pneumonia, inappropriate antimicrobial treatment, recent surgery, mechanical ventilation, acute renal failure, septic shock, and DIC^[^^14^^,^^17^^,^^22^^,^^23^^]^, the last three of which also found to be statistically significant in our study. 

 The emergence of XDR *A. baumannii* is a therapeutic problem world over. Studies have shown that the antibiotic susceptibility rates for Acinetobacter have decreased over the years^[^^13^^]^. The known resistance mechanisms of *A. baumannii* to antimicrobials are the production of broad-spectrum β-lactamases, aminoglycoside-modifying enzymes, and changes in outer membrane porins and alterations in penicillin-binding proteins (PBP)^[^^24^^]^. For MDR/XDR infections in resource-limited settings, the only available therapeutic option is polymyxin^[^^12^^]^. Tigecycline, a glycylcycline antibiotic, has good in vitro activity against PRA, but is very costly and not readily available in developing countries^[^^24^^]^.

 A very recent (prospective, multicenter) American study of nosocomial blood stream infections due to Acinetobacter species (including *A. baumannii*) showed only 10% multi-drug resistance while the overall susceptibility for carbapenems was as high as 95.2%^[^^25^^]^. In contrast to this, carbapenems showed only 5.4% sensitivity in our study and colistin found to be the most effective drug, being sensitive in 95% of cases. PDR is being increasingly reported^[^^26^^,^^27^^]^ and therefore to confront the imminent threat of untreatable infection caused by this organism, an appropriate antibiotic strategy should be addressed, and strict compliance with basic and potential control measures for the containment of infection should be instituted.

 We believe that our study adds information regarding XDR *A. baumannii* infection in critically ill children. Large multi-centric randomized controlled trials are needed for further risk stratification of this bacterium. Limitations of this study are that since this is a retrospective study we were not able to evaluate all variables. 

Prospective studies are warranted.

## Conclusion

The present study is unique in evaluating risk factors as well as predictors of mortality among critically ill children with XDR mono-microbial infection caused by *A. baumannii*. The high resistance rates found in this study may be associated with the high frequency at which these antimicrobial drugs are used for both prophylactic and therapeutic treatment of hospitalized children. Continuous bacteriological surveillance, early diagnosis, rational use of antibiotics to prevent drug resistance, and strict use of infection control policies are required.
